# A Risk Model Composed of Complete Blood Count, *BRAF V600E* and *MAP2K1* Predicts Inferior Prognosis of Langerhans Cell Histiocytosis in Children

**DOI:** 10.3389/fonc.2022.800786

**Published:** 2022-02-04

**Authors:** Leyuan Wang, Lin Yuan, Xizi Du, Kai Zhou, Yu Yang, Qingwu Qin, Liangchun Yang, Yang Xiang, Xiangping Qu, Huijun Liu, Xiaoqun Qin, Chi Liu

**Affiliations:** ^1^ Department of Physiology, School of Basic Medicine Science, Central South University, Changsha, China; ^2^ Department of Respiratory and Critical Care Medicine, The Second Xiangya Hospital, Central South University, Changsha, China; ^3^ Department of Pediatrics, Xiangya Hospital, Central South University, Changsha, China; ^4^ Research Center of China-Africa Infectious Diseases, Xiangya School of Medicine Central South University, Changsha, China

**Keywords:** Langerhans cell histiocytosis, complete blood counts, *BRAF V600E*, *MAP2K1*, prognosis

## Abstract

**Background:**

In children, Langerhans cell histiocytosis (LCH), which is the most prevalent histiocytic disorder, exhibits a wide variety of manifestations and outcomes. There is no standard prognosis evaluation system for LCH. We investigated the combined predictive significance of complete blood counts (CBCs), *BRAF V600E* and *MAP2K1* in childhood LCH.

**Methods:**

A cohort of 71 childhood LCH patients was retrospectively studied. The prognosis predictive significance of platelet-to-lymphocyte ratio (PLR), neutrophil-to-lymphocyte ratio (NLR), systemic inflammation response index (SIRI), systemic immune inflammation index (SII), *BRAF V600E*, and *MAP2K1* were analyzed.

**Results:**

Histiocyte Society (HS) classification of LCH patients was correlated with NLR, SIRI, and progression free survival (PFS), bone involvement was correlated with SIRI, liver involvement was correlated with NLR, SII, SIRI, and PFS, spleen involvement was correlated with SIRI, lung involvement was correlated with NLR and PFS, CNS involvement was correlated with PFS, while *BRAF V600E* was correlated with PLR, NLR, SIRI, SII, PFS, and OS (*p* <0.05). *MAP2K1* was correlated with NLR, SIRI, PFS, and OS (*p* <0.05). Elevated NLR, PLR SIRI, and SII predicted inferior PFS and OS (*p <*0.05). PLR, NLE, SIRI, SII, *BRAF V600E*, and *MAP2K1* were used to establish a risk model for stratifying the LCH patients into 3 different risk groups. Respective median PFS for low-, mediate-, and high-risk groups were not reached, 26, and 14 months (*p* <0.001), and all median OS were not reached (*p* <0.001).

**Conclusion:**

The risk model combined with CBCs, *BRAF V600E*, and *MAP2K1* might be a promising prognostic system for LCH in children.

## Introduction

Langerhans cell histiocytosis (LCH), also referred to as histiocytosis, defines a collection of histiocytosis disorders of unknown cause. Clinically, LCH exhibits various manifestations, from single indolent lesions to explosive multisystem diseases (1). LCH can develop at any age, with a highest incidence in children aged 1–3 years old. Although the prognoses of LCH patients have slightly improved in the past decades, the standard treatment regimen fails to cure more than 50% patients with the multisystem disease (2). Prognostic prediction depends on whether risk organs (liver, bone, and spleen) are involved at the time of diagnosis, and response to initial therapy (3). However, there is no widely accepted prognosis evaluation system for LCH.

In recent years, the improvement of genomic technology leads to a deep understanding of LCH. In 2010, Badalian-Very et al. (4) reported that about 57% LCH patients had *BRAF V600E* mutation for the first time. *BRAF V600E*, a vital kinase of the RAS–RAF–MEK–ERK signal-transduction pathways, has several functions. Apart from *BRAF V600E* mutations, various activating mutations, such as *MAP2K1* mutation, have been reported in LCH (5). Previous studies reported that *BRAF V600E* mutation may be closely related to multisystem diseases and poorer prognosis (6), and *MAP2K1* mutation was related to risk organ involvement (7). With the optimization of LCH treatment regimen, the understanding of genotype needed to be deeper.

Systemic inflammatory responses and host immunity are closely associated with cancer development and prognostic outcomes (8). Complete blood counts (CBC) combined with each other, such as platelet-to-lymphocyte ratio (PLR), neutrophil-to-lymphocyte ratio (NLR), systemic inflammation response index (SIRI), and systemic immune inflammation index (SII), can be used as prognostic predictors for cancers (9, 10). However, the significance of CBCs in LCH should be evaluated further.

We investigated the predictive values of CBCs, *BRAF V600E*, and *MAP2K1* in 71 childhood LCH, and evaluated a new risk model that combined with CBCs, *BRAF V600E*, and *MAP2K1* for prognostic prediction of childhood LCH patients.

## Methods

### Study Participants

We used Cochran formula and PASS software to calculate the sample size. Seventy-one childhood LCH patients at the Xiangya Hospital, Central South University were retrospectively evaluated. These patients were diagnosed from January 2014 to December 2020. The inclusion criteria were: i. Pathologically confirmed LCH and ii. Age ≤14 years. The exclusion criteria were: i. Patients administered with radiotherapy, chemotherapy or any other therapies prior to diagnosis; ii. An autoimmune disease history; iii. A chronic inflammatory disease history, including inflammatory bowel diseases; and iv. Uncontrolled infections that are active or the presence of other illnesses.

The 71 LCH patients had their complete follow-up and clinical data. Patient follow-up had been done from the diagnostic day to June 2021, and there was no patient loss to follow-up. The Helsinki Declaration of 1975, which was revised in 2008 was adhered to during this study.

### Identification of BRAF V600E and MAP2K1 Mutation

DNA from the paraffin-fixed LCH lesion tissue was extracted by DNA extraction kit (product of QIAGEN, Cat NO.56404) after histologic review by ≥10% histiocytes. *BRAF V600E* mutations were evaluated by droplet digital polymerase chain reaction (dd-PCR) *via* the Raindrop system (Raindance Technologies, Billerica, CA) (11). DNA samples were collected from peripheral blood cells, and whole-exome sequencing analysis was performed to detect *MAP2K1* mutation status (12).

### Data Collection

Clinical data, namely, gender, age, Histiocyte Society (HS) classification, organ involvement, and treatment efficacy after a 6-week induced treatment that conducted LCH-III protocol—vinblastine and prednisone (13), were obtained. Routine blood analysis was done within a week prior to therapeutic initiation. Various parameters, such as PLR, NLR, SIRI, and SII were determined as: PLR = platelet counts/lymphocyte counts, NLR = neutrophil counts/lymphocyte counts, SIRI = neutrophil counts × monocyte counts/lymphocyte counts, SII = platelet counts × neutrophil counts/lymphocyte counts. A risk model with the above 4 CBC variables, *BRAF V600E*, and *MAP2K1* was evaluated as: i. A score of 1 (low-risk) denoted no mutation of *BRAF V600E* or *MAP2K1*, and elevated expression of 0–2 CBCs, ii. A score of 2 (mediate-risk) denotes no mutation of *BRAF V600E* or *MAP2K1*, and high expression of 3–4 CBCs; 1 mutation of *BRAF V600E* or *MAP2K1*, and high expression of 0–2 CBCs; both of *BRAF V600E* and *MAP2K1* were mutated, and high expression of 0–1 CBCs (3), 1 mutation of *BRAF V600E* or *MAP2K1*, and high expression of 3–4 CBCs; both of *BRAF V600E* and *MAP2K1* were mutated, and high expression of 2–4 CBCs. Overall survival (OS) is the time length between diagnostic date to the date of death due to any cause or to last follow-up date. Progression free survival (PFS) denotes the time length from diagnostic date to dates of death or disease progression.

### Statistical Analysis

The SPSS 22.0 software (SPSS Inc., Chicago, IL, USA) was employed in all statistical analyses. Associations between NLR, PLR, SIRI, SII, and clinico-pathological features for LCH patients were assessed by the Pearson’s χ^2^ test. The CBCs cut-off thresholds were determined by receiver operating characteristic (ROC) curves. Survival curves were established by the Kaplan–Meier method, and were compared by the log-rank test. The threshold for significance was p ≤0.05.

## Results

### Patient Characteristics

**Table 1** shows clinico-pathological features of 71 childhood LCH patients. Among the 71 patients, 38 (53.5%) were male while females accounted for 33 (46.5%). The median age was 4-years-old. As for the HS classification, 41 (57.8%) were single-system involvement (SS), 14 (19.7%) were multi-systemic involvement minus risk organ involvement (MS RO−), 16 (22.5%) were multiple system involvement with one or more risk organs involvement (MS RO+). As for organ involvement, 5 (7%) presented with bone involvement, 14 (19.7%) with liver involvement, 4 (5.6%) with spleen involvement, 7 (9.9%) with lung involvement and 27 (38%) with central nervous system (CNS) involvement. A total of 19 (26.8%) patients presented with *BRAF V600E* mutation, 52 (73.2%) presented with *BRAF V600E* wild type. Five (7%) patients presented with *MAP2K1* mutation, 66 (93%) presented with *MAP2K1* wild type. After a 6-week treatment, 8 (11.3%) were evaluated non-active disease (NAD), 35 (49.3%) active disease-better (AD-better), 12 (16.9%) active disease-stable (AD-stable), while 16 (22.5%) active disease-progressive (AD-progressive).

**Table 1:** Clinicopathological characteristics of patients (n = 71).

Characteristics	Number (%)
Gender	
Male	38 (53.5%)
Female	33 (46.5%)
Age	
Median age at diagnosis	4-year-old
HS classification	
SS	41 (57.8%)
MS RO−	14 (19.7%)
MS RO+	16 (22.5%)
Involvement	
Bone	5 (7%)
Liver	14 (19.7%)
Spleen	4 (5.6%)
Lung	7 (9.9%)
CNS	27 (38%)
*BRAF V600E*	
Mutation	19 (26.8%)
Wild type	52 (73.2%)
*MAP2K1*	
Mutation	5 (7%)
Wild type	66 (93%)
Treatment efficacy after 6-week treatment	
NAD	8 (11.3%)
AD-better	35 (49.3%)
AD-stable	12 (16.9%)
AD-progressive	16 (22.5%)

### Optimal Thresholds for CBCs and Their Associations With Clinico-Pathological Features

**Figure 1:**
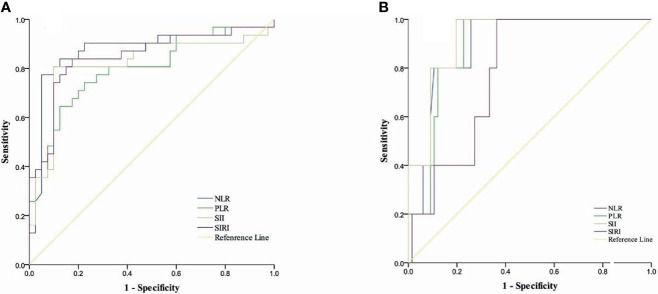
Cut-off thresholds for CBCs. **(A)** ROC curves for ideal cut-off thresholds of NLR, SII, PLR, and SIRI for PFS. **(B)** Roc curves for ideal cut-off thresholds of NLR, PLR, SII, and SIRI for OS.

ROC curves were used to establish optimal thresholds for CBCs. **Figure 1A** shows that the optimal PFS thresholds for NLR, PLR, SII, and SIRI were 2.69 (area under curve (AUC) = 0.875, sensitivity = 0.774, specificity = 0.95), 221.88 (AUC = 0.798, sensitivity = 0.645, specificity = 0.875), 802.89 (AUC = 0.817, sensitivity = 0.806, specificity = 0.90), and 1.15 (AUC = 0.847, sensitivity = 0.839, specificity = 0.825), respectively. **Figure 1B** shows that optimal thresholds for OS were 2.92 (AUC = 0.895, sensitivity = 1, specificity = 0.258), 257.81 (AUC = 0.891, sensitivity = 1, specificity = 0.773), 1143.46 (AUC = 0.924, sensitivity = 1, specificity = 0.803), and 1.46 (AUC = 0.782, sensitivity = 1, specificity = 0.636) for NLR, PLR, SII, and SIRI, respectively.

As for the clinico-pathological features of the LCH patients, HS classification was correlated with the levels of NLR and SIRI, bone involvement was correlated with SIRI, liver involvement was correlated with NLR, SII and SIRI, spleen involvement was correlated with SIRI, and lung involvement was correlated with NLR (*p* <0.05). Gender or age of the patients was not correlated with CBCs (*p* >0.05; **Table 2**).

**Table 2:** Clinico-pathological characteristics according to CBCs.

Characteristics	NLR high	NLR low	*p*-value	PLR high	PLR low	*p*-value	SII high	SII low	*p*-value	SIRI high	SIRI low	*p*-value
Gender			0.967			0.85			0.801			0.872
Male	14	24		13	25		15	23		18	20	
Female	12	21		12	21		14	19		15	18	
Age			0.066			0.197			0.126			0.083
Median age at diagnosis												
HS classification			**0.024**			0.67			0.308			**0.031**
SS	10	31		14	27		14	27		15	26	
MS RO−	6	8		4	10		6	8		6	8	
MS RO+	10	6		7	9		9	7		12	4	
Involvement												
Bone	3	2	0.26	2	3	0.816	3	2	0.366	5	0	**0.013**
Liver	9	5	**0.016**	7	7	0.196	9	5	**0.046**	12	2	**0.001**
Spleen	2	2	0.567	1	3	0.66	2	2	0.701	4	0	**0.027**
Lung	5	2	**0.044**	2	5	0.698	3	4	0.909	5	2	0.163
CNS	13	14	0.114	10	17	0.801	13	14	0.327	15	12	0.23
*BRAF V600E*			**0.001**			**0.016**			**0.001**			**0.001**
Mutation	13	6		11	8		14	5		15	4	
Wild type	13	39		14	38		15	37		18	34	
*MAP2K1*			**0.037**			0.229			0.065			**0.013**
Mutation	4	1		3	2		4	1		5	0	
Wild type	22	44		22	44		25	41		28	38	

p value < 0.05 is listed as bold number.

### Correlation Between Gene Statuses of BRAF V600E, MAP2K1, and CBCs

As shown in **Table 2**, *BRAF V600E* mutations were correlated with levels of NLR, SIRI, PLR, and SII, while *MAP2K1* mutation was associated with NLR and SIRI (*p* <0.05).

### Prognostic Factors

At follow-up, 31 patients were found to exhibit disease progression. Median PFS was 13 months, 5 patients died, while the median OS was 25 months. As shown in **Table 3**, HS classification, involvement of liver, lung and CNS were unfavorable PFS predictors (*p* <0.05), whereas gender, age, HS classification or organ involvement had no correlation with OS (*p* >0.05).

**Table 3:** Correlation between clinico-pathological characteristics and prognostic outcomes.

Characteristics	Median PFS (months)	*p*-value	Median OS (months)	*p*-value
Gender		0.763		0.281
Male	31		Not reached	
Female	27		Not reached	
Age		0.072		0.932
Median age at diagnosis	14		Not reached	
HS classification		**0.001**		0.693
SS	Not reached		Not reached	
MS RO−	36		Not reached	
MS RO+	10		Not reached	
Involvement				
Bone	12	0.084	Not reached	0.213
Liver	10	**<0.001**	Not reached	0.269
Spleen	3	0.128	Not reached	0.143
Lung	12	**0.011**	Not reached	0.635
CNS	24	**0.027**	Not reached	0.248
*BRAF V600E*		**0.008**		**0.003**
Mutation	17		Not reached	
Wild type	Not reached		Not reached	
*MAP2K1*		**0.017**		**<0.001**
Mutation	5		Not reached	
Wild type	36		Not reached	

p value < 0.05 is listed as bold number.

**Figure 2:**
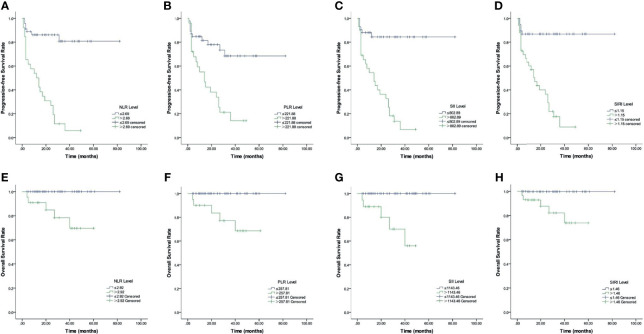
Kaplan–Meier survival curves of LCH patients. **(A–D)** Kaplan–Meier curves for PFS according to NLR **(A)**, PLR **(B)**, SII **(C)** and SIRI **(D)**. **(E–H)** Kaplan–Meier curves for OS according to NLR **(E)**, PLR **(F)**, SII **(G)**, and SIRI **(H)**.

**Figure 3:**
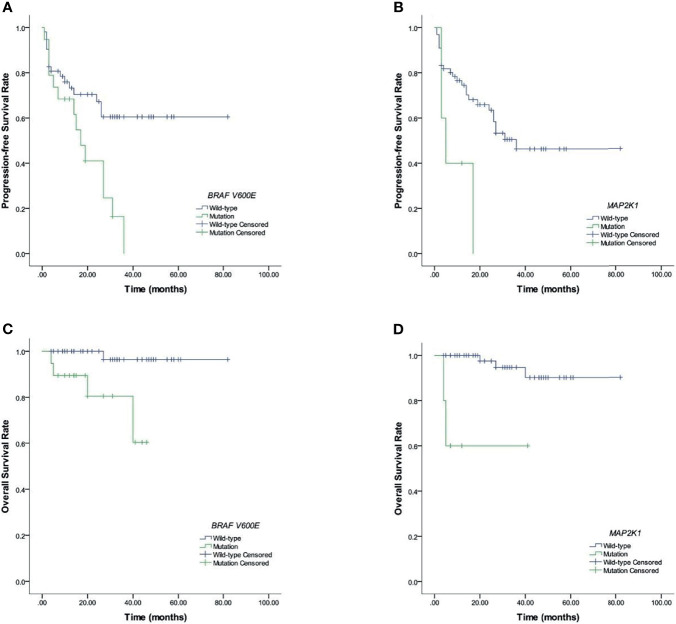
Prognostic *BRAF V600E* and *MAP2K1* in LCH patients. **(A, B)** Results for PFS. **(C, D)** Results for OS.

**Figures 2**, **3** and **Tables 3**, **4** show the survival curves for OS and also PFS with respect to *BRAF V600E*, CBCs and *MAP2K1* are presented in. **Figures 2A–D** show that elevated: PLR (*p* <0.001), SII (*p* <0.001), NLR (*p* <0.001), and SIRI (*p* <0.001) were inferior PFS predictors. **Figures 2E–H** show that high: NLR (*p* = 0.002), PLR (*p* = 0.002), SII (*p* <0.001), and SIRI (*p* = 0.01) were inferior OS predictors. *BRAF V600E* (*p* = 0.008) and *MAP2K1* (*p* = 0.017) mutations were predictive factors for inferior PFS, and *BRAF V600E* (*p* = 0.003) and *MAP2K1* (*p* <0.001) mutations were inferior OS predictor markers (**Figure 3**).

**Figure 4:**
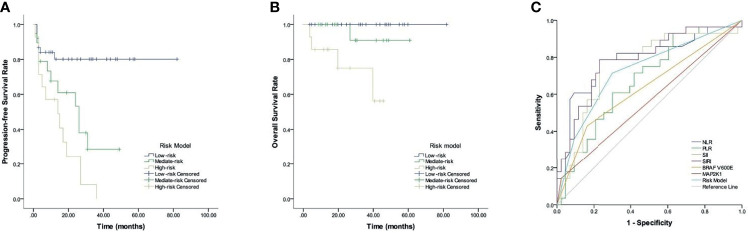
Prognostic risk model in LCH patients. **(A)** PFS. **(B)** OS. **(C)** ROC curves analysis for optimal cut-off value of treatment efficacy.

### Prognostic Significance of the Newly Risk Model

Given the predictive values of CBCs, *BRAF V600E*, and *MAP2K1* for LCH, we established a new risk model incorporated NLR, PLR, SII, SIRI, *BRAF V600E*, and *MAP2K1*, and evaluated its prognostic value. **Table 4** and **Figure 4** show that LCH patients were allocated into 3 different groups based on the risk model: low-, mediate-, and high-risk groups. Respective median PFS for low-, mediate-, and high-risk groups were not reached, 26, and 14 months (*p* <0.001), and all median OS were not reached (*p* = 0.002).

**Table 4:** Univariate analysis of CBCs and risk model associated with PFS and OS.

Variates	Median PFS (months)	*p*-value	Median OS (months)	*p*-value
NLR		**<0.001**		**0.002**
High	12		Not reached	
Low	Not reached		Not reached	
PLR		**<0.001**		**0.002**
High	14		Not reached	
Low	Not reached		Not reached	
SII		**<0.001**		**<0.001**
High	14		Not reached	
Low	Not reached		Not reached	
SIRI		**<0.001**		**0.01**
High	15		Not reached	
Low	Not reached		Not reached	
Risk model		**<0.001**		**0.002**
Low-risk	Not reached		Not reached	
Mediate-risk	26		Not reached	
High-risk	14		Not reached	

p value < 0.05 is listed as bold number.

We analyzed the predictive ability of the risk model in distinguishing treatment efficacy after 6-week treatment. ROC curve analysis results showed that the AUC values of NLR, PLR, SII, SIRI, *BRAF V600E*, *MAP2K1* and risk model were 0.784 (*p* <0.001), 0.675 (*p* = 0.013), 0.752 (*p* <0.001), 0.786 (*p* <0.001), 0.633 (*p* = 0.006), 0.56 (*p* = 0.043), and 0.727 (*p* = 0.001), respectively.

## Discussion

LCH is a histiocytosis disease described by aberrant cellular functions, proliferation or differentiation of mononuclear phagocyte system cells (14). LCH is clinically heterogeneous, ranging from single system involvement to multiple system involvements. The outcome of LCH is highly variable. Therefore, finding prognostic factors for LCH patients is an urgent need. We evaluated the prognostic importance of CBCs, *BRAF V600E*, and *MAP2K1* in childhood LCH, and combined them to build a new risk model. Elevated NLR, PLR, SII, *BRAF V600E*, SIRI, and *MAP2K1* mutations predicted inferior PFS, OS and worse treatment efficacy after a 6-week treatment, and the new risk model could be used for stratifying patients with LCH into various risk categories for prognostic prediction.

LCH was initially considered an immune-dysregulatory disorder rather than a neoplastic disorder, until somatic activation of gene mutations in the mitogen-activated protein kinase (MAPK) pathway were found in LCH patients, and reclassified LCH to be a myeloid neoplastic disorder (4, 15). About 60% of the LCH patients presented with *BRAF V600E* mutations, and other genetic alterations that activated the MAPK pathway including *MAP2K1* (16). Thus, clinical responses are targeted to BRAF or MEK1 (the product of the *MAP2K1* gene) inhibitors which have been observed in LCH with *MAP2K1* and *BRAF V600E* mutations (17). The *BRAF V600E* mutation is correlated with worse prognostic outcomes in several cancer types. In metastatic colorectal cancers, patients with *BRAF V600E* mutation had a shorter OS than *BRAF V600E* wild type (18). In pediatric low-grade glioma, patients harboring the *BRAF V600E* mutation exhibited poor clinical outcomes after chemotherapy and radiotherapy with a 10-year PFS rate of 27% and 60.2% for the *BRAF V600E* wild type (19). *MAP2K1* mutation is a rare oncogenic alteration, and is reportedly associated with the development and prognosis of cancers (20). In advanced colorectal cancer, all the patients with *MAP2K1* mutation treated with anti-EGFR, anti-EGFR combined with MEK and BRAF inhibitors, or anti-EGFR combined with ERK inhibitors, showed disease progression, where the *MAP2K1* mutation was associated with poor response to targeted therapy (21). *MAP2K1* mutation was also found in splenic diffuse red pulp small B-cell lymphoma, and it was related to an aggressive disease, and the PFS of *MAP2K1* mutation patients were shorter than the wild type one (22). In the present study, we found that 26.8% childhood LCH patients presented with *BRAF V600E* mutations, 7% presented with *MAP2K1* mutation, where *BRAF V600E* and *MAP2K1* mutations were predictors of inferior PFS, OS and worse treatment efficacy after a 6-week treatment. Our result was consistent with the previous findings.

Alterations in the surrounding environment could influence the development and progression of tumors. Secretion of various cytokines, chemokines, changes of immune cells, and other factors can affect the tumor microenvironment (TME) (23). Immune cells, such as innate immune cells (neutrophils, NK cells, lymphocytes, dendritic cells, and macrophages) and adaptive immune cells (T and B cells), play important roles in TME. CBCs made up of neutrophils, monocytes lymphocytes, and plates, are reportedly promising in predicting prognosis of several types of cancer. Combined CBCs of the above variates, namely, NLR, SIRI, PLR, and SII are potential markers in prognosis prediction of cancers. In breast cancer, low PLR and NLR are considerably correlated with delayed metastasis, while metastasis-free survival was superior in these patients (24). High preoperative and postoperative NLR, particularly persistently elevated pre- to post-operative NLR are prognostic markers for poor OS in gastric cancer patients (25). In hepatocellular carcinoma, high SII predicted shorter recurrence-free survival and OS (26). In advanced lung adenocarcinoma treated with first-generation EGFR-TKIs, elevated SIRI was correlated with worse ECOG PS, EGFR 19-Del mutation, OS and PFS, SIRI was an independent survival predictor (27). The pathological characteristic of LCH lesions is a vigorous inflammatory cells infiltrate surrounding pathological histiocytes. Little is known about the effect of these inflammatory cells on LCH. Our result showed that high NLR, SIRI PLR, and SIRI predicted inferior PFS, OS and worse treatment efficacy after a 6-week treatment, which is consistent with the previous findings.

We established a new risk model that incorporated NLR, PLR, SII, SIRI, *BRAF V600E* and *MAP2K1*, and assessed its prognostic significance in childhood LCH. The respective median PFS for low-, mediate-, and high-risk groups were not reached, 26 and 14 months, and all the median OS were not reached. We also found that the risk model could predict treatment efficacy after a 6-week treatment. Feng et al. (28) conducted a CBCs model which consisted of NLR, lymphocyte–monocyte ratio, and PLR. They established that the respective median PFS for low-, intermediate-, and high-risk groups were not reached, 16 and 7 months, while respective median OS for the 3 groups were not reached, 46 and 20 months. Our previous study also established a CBCs model incorporating NLR, PLR, SII, and SIRI for primary central nervous system lymphoma, which accurately predicted the median PFS and median OS (10). Findings of this study are consistent with the previous findings, indicating that the new risk model could be used for prognosis prediction in childhood LCH.

In summary, our study showed that the new risk model combined with CBCs, *BRAF V600E*, and *MAP2K1* might be a promising prognostic system for LCH in children. However, some limitations associated with the present study are worth mentioning. First, we enrolled a small sample size at a single center. Second, the CBCs can be influenced by a variety of factors, including immune status that may bias the findings. Studies should be conducted to verify the roles of CBCs, *BRAF V600E*, and *MAP2K1* in childhood LCH.

## Data Availability Statement

The original contributions presented in the study are included in the article/supplementary material. Further inquiries can be directed to the corresponding author.

## Ethics Statement

The studies involving human participants were reviewed and approved by the Ethics Committee of Xiangya Hospital of Central South University. Written informed consent to participate in this study was provided by the participants’ legal guardian/next of kin.

## Author Contributions

LW and CL designed the research study and collected the clinical data. LinY, XD, KZ, YY performed the research. QQ, LiaY and YX and drafted the manuscript. XQu, HL, and XQi participated in the literature search and analyzed the data. All authors listed have made a substantial, direct, and intellectual contribution to the work and approved it for publication.

## Funding

The study was supported by funds from the NSFC under the following grant numbers: #81600026, #31900424, #81970033, #82070034; funding from the Open Foundation of Hunan College Innovation Program (grant number #20K142), funds from the Hunan Natural Science Foundation under grant numbers #2019JJ50760, #2020JJ4688, and #2020JJ4776, and funding from the Open Sharing Fund for the Lager-scale Instruments and Equipment of Central South University.

## Conflict of Interest

The authors declare that the research was conducted in the absence of any commercial or financial relationships that could be construed as a potential conflict of interest.

## Publisher’s Note

All claims expressed in this article are solely those of the authors and do not necessarily represent those of their affiliated organizations, or those of the publisher, the editors and the reviewers. Any product that may be evaluated in this article, or claim that may be made by its manufacturer, is not guaranteed or endorsed by the publisher.
